# Sonogram of coccygeus muscle in dairy cows with different gestational ages

**DOI:** 10.1186/s40781-017-0152-6

**Published:** 2017-12-18

**Authors:** Mokhamad Fakhrul Ulum, Dilla Frastantie, Bambang Purwantara

**Affiliations:** Department of Clinic Reproduction and Pathology, Faculty of Veterinary Medicine, Bogor Agricultural University, Jalan Agatis Kampus IPB Dramaga, Bogor, Jawa Barat 16680 Indonesia

**Keywords:** Ultrasonography, Coccygeus muscle, Thickness, Intensity, Gestation age and postpartum

## Abstract

**Background:**

The change in size and weight of the female reproductive organs during gestation and birth might be affect the perineal muscles and this condition in dairy cow not been reported. This study aimed to assess the ultrasonographic image of coccygeus muscle in 11 inseminated dairy cows with different gestational ages and postpartum.

**Methods:**

Gestational age was calculated based on the record of artificial insemination and confirmed by using transrectal brightness mode ultrasonography. Perineal hair between the sacrum and ischium bones was shaved along 3–5 cm before being ultrasound. The images of perineal area were obtained by transcutaneous ultrasound using a 5.0 MHz transducer. The thickness and intensity of the coccygeus muscle were measured and analyzed by gestational status and postpartum to show the differences.

**Results:**

The results showed that the thickness of coccygeus muscle increased with the increase in gestational age. Muscle intensity only increased at young gestational age. However, it decreased with the increase in gestational age (*P* < 0.05).

**Conclusions:**

The ultrasound image of coccygeus muscle was affected by gestational status, thus this method may be used as one of the new methods of indirect gestational detection on dairy cows.

## Background

The reproductive cycle is a process of reproduction that takes place through multiple reproductive activities in order to produce offsprings. Estrous, mating, gestation, and birth are a series of reproductive activities in a reproductive cycle [1]. Based on the clinical signs, estrous in dairy cows are characterized by vulva swelling, ruddy vulva, and slimy vagina [2]. Estrous is also characterized by moving its tail aside to make it easier for bulls to copulate during the mating process [1]. During the mating process, fertilization will occur and fetus will be formed in the uterus that develops from a size of several grams to few kilograms [3]. The reproductive cycle ends with birth or partus where fetus in the uterus is born, producing an independent individual.

Reproductive activities alter the body’s physiology dynamically on activities that are not visible can be monitored by various methods. Muscular and skeletal systems that are affected by gestational status can be monitored manually or by radiological tools. Radiological monitoring can be performed using X-ray (i.e. radiography, computed tomography, and fluoroscopy) for bone-tissues and ultra high frequency sound (i.e. ultrasonography) for soft tissues such as muscles and internal organs [4]. Monitoring of gestational status in dairy cows can be performed using various methods. The simplest and easiest method is by perectal palpation. However, this method can only be performed at least >35 days gestation by skilled workers [5]. Gestational detection in dairy cows with young gestational age around 12 days is difficult to be performed by perectal palpation. Detection of young gestation in dairy cows is usually performed by transrectal ultrasonography. Ultrasonographic monitoring of the changes in abdominal muscle tissue size of Ettawa grade does differ in size with different gestational ages [6]. Measurement of pelvic bone by Computed Tomography (CT) on dairy cows showed change in size with age that was useful as a prediction for calving difficulties [7]. CT image in Estonian native and crossbreed cattle also showed a difference in size on various parameters [8].

Anatomically, the female reproductive organs attach to the base of the vulva as the orifice of the vagina. The vagina is a reproductive tract supported by the perineal muscle comprising of piriformis, coccygeus, iliococcygeus, and pubococcygeus muscles to maintain its stability [9]. The base (caudal) of the vagina is supported by the levator ani muscle of iliococcygeus and pubococcygeus [10]. The change in size and weight of the female reproductive organs during gestation and birth will affect the contraction and relaxation of the muscles. This condition causes the amount of load supported by the tissues where the reproductive organs are attached to change following the anatomical changes. The changes in these muscles in cows with different gestational status and postpartum have not been reported. CT is an imaging diagnostic tools which fit to depict harder tissues i.e. bone, tumor, blood cloth. However, the CT cannot distinguish parts of soft tissues while the diagnostic ultrasonography may cover both hard and soft tissue imaging. CT is an ionizing radiation diagnostic tool and to be a contraindication for pregnant animal and fetus. But, ultrasound is a non-ionizing radiation and considered as a safe diagnostic for obstetrical and gynecological purposes. Ultrasonography was used in this study to investigate the anatomic changes and the intensity of coccygeus muscle with the size and weight of the uterus that increases with gestational age and decreases after postpartum in dairy cows.

## Materials and methods

### Materials

This research has obtained the approval from Animal Ethic Commission of Faculty of Veterinary Medicine, Bogor Agricultural University with certificate number: 048 / KEH / SKE / XI / 2015. A total of 11 dairy cows of Friesian Holstein descendants from Sentra Peternakan Rakyat (SPR) breeders in Kawasan Usaha Peternakan (KUNAK) Cibungbulang, Bogor, West Java of Indonesia, were used in this study. The services were given by artificial insemination (AI) as recorded in the enclosure record. Gestational status was determined based on the last record of insemination and confirmed by transrectal B-mode ultrasonography.

### Monitoring of post-insemination gestation based on ultrasonography

Gestational status was confirmed by transrectal B-mode ultrasonography. Dairy cows were restrained in a communal cowshed using a nasal rope in standing position. Ultrasonography scanning was performed by using 4VetMini ultrasonography console (Draminski, PT Agroprima Lab, Indonesia). The transducer used was linear type with frequency of 5.0 MHz. Lubricant gel was used to facilitate the process of inserting the hand and transducer into the rectum. The gestational status on the ultrasonography display was documented and saved in JPEG format for further analysis.

### Ultrasonographic image of the coccygeus muscle

Transcutaneous B-mode ultrasonography scanning of coccygeus muscle was performed using the same ultrasonography console and transducer immediately after gestational status confirmation. Perineal hair between sacrum and ischium bones was shaved along 3–5 cm. Ultrasound gel was applied onto the surface of the skin to produce good quality sonogram. The scanning angles used were vertical and horizontal to the axis of the body of dairy cows. Ultrasonographic images of the perineal tissue layers between the sacrum and ischium bones were further documented and saved in JPEG format. The sonogram display of perineal constituent tissues were observed and identified layer by layer.

### Sonographic image analysis

The thickness (mm) of the constituent tissues of perineum in sonogram were then measured using an image processing software (ImageJ, NIH, USA). Gray color intensity of the muscle tissue from the sonogram was then measured in arbitrary unit (a.u.) and further analyzed using the same software.

### Data analysis

The data obtained were analyzed statistically using SPSS v.16.0 (SPSS Inc., Chicago, IL) with Oneway-ANOVA post hoc Duncan test to determine the differences between groups with a significance level of *P* < 0.05. The data were presented in the form of means with standard deviation (mean ± SD).

## Results

Table 1 showed the artificial insemination record (AI record) and confirmation result by ultrasonography examination on dairy cows. There was a difference in percentage of gestation on the dairy cows based on the AI record and confirmation by ultrasonography. The ultrasonography results showed a lower percentage of gestation (63.64%) than the AI record results (81.82%) in dairy cows.

**Table 1:** Gestational status of dairy cows in artificial insemination (AI) record and confirmation of transrectal ultrasononography examination

No	Groups	AI record		Ultrasononography		
		Number (n)	Gestation (%)	Number (n)	Gestation (%)	Sonogram
1	Non-pregnant	2	18.18	4	36.36	Empty uterine
2	Pregnant <1 month (8–24 days)	5	45.45	3	27.27	Gestational sac in uterine
3	Pregnant 2–3 months (70–90 days)	3	27.27	3	27.27	Placenta and fetal in uterine
4	Postpartum 2 week (12–14 days)	1	9.10	1	9.10	Lochia in uterine
	Total	11	100.00	11	100.00	

**Fig. 1:**
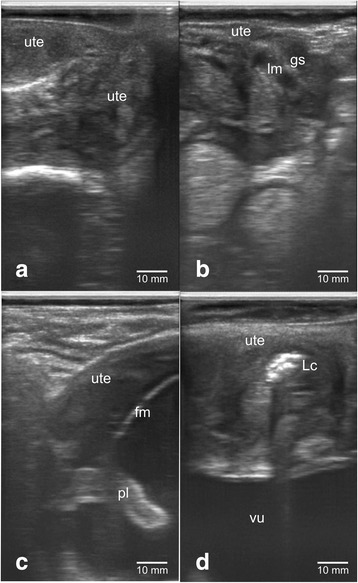
Sonogram of dairy cows with different gestational ages in transrectal B-mode ultrasonography: **a** non-pregnant, **b** <1 month pregnant, **c** 2–3 months pregnant, **d** postpartum. Note: ute = uterus, lm = lumen, gs = gestational sac, fm = fetal membrane, pl = placenta, Lc = lochia, vu = bladder

Figure 1 showed the confirmation results of dairy cow's gestational status using transrectal B-mode ultrasonography. The uterus of non-pregnant cow appeared to be groovy with folds uterine endometrium on the inside (Fig. 1a). Dairy cow’s uterus of <1 month (8–9 days) pregnancy showed mucus-filled uterine lumen with the presence of gestational sac that appeared as anechoic (Fig. 1b). Fetus was seen inside the pregnant dairy cow’s uterus but not all body parts can be seen on a sonogram screen. The fetal and placenta membranes were seen as hyperchoic, while the fluid inside appeared as anechohic (Fig. 1c). The uterus during postpartum contained lochia which was excreted during uterine involution with echogenicity that varies from anechoic (black color) to hyperechoic (white color) (Fig. 1d).

Table 2 showed measurements of each layer of tissue that constitutes the perineal area between the sacrum and ischium bones of dairy cows, and the intensity of the coccygeus muscle. The thickness of tissue in general showed no significant difference among dairy cows with different gestational ages (*P* > 0.05). However, when observed in detail, the thickness of the skin and subcutaneous tissue slightly increased with the increase of gestational age. The muscle and total tissue thickness also increase in higher rate than that skin and subcutaneous tissue. Muscle intensity showed an increase in young gestational age, but subsequently decreased with an increase in gestational age and postpartum which differed statistically among the groups of dairy cows (*P* < 0.05).

**Table 2:** Biometry and tissue intensity of the perineal area with different gestational status and postpartum

No	Observations	View	Group				*P*-value
			TB (*n* = 4)	B < 1 (n = 3)	B2–3 (*n* = 3)	PP* (*n* = 1)	
1	Skin (mm)	H	4.6 ± 0.2^a^	4.7 ± 0.2^a^	4.6 ± 0.2^a^	4.7	0.65
		V	3.9 ± 1.1^a^	4.1 ± 0.3^a^	4.3 ± 0.2^a^	5.1	0.80
2	Subcutaneous (mm)	H	1.6 ± 0.4^a^	2.2 ± 0.6^a^	2.1 ± 0.5^a^	2.1	0.23
		V	1.8 ± 0.6^a^	2.4 ± 0.4^a^	2.3 ± 0.9^a^	2.1	0.45
3	Muscle (mm)	H	19.5 ± 4.4^a^	19.5 ± 5.3^a^	22.8 ± 10.4^a^	21.3	0.79
		V	16.1 ± 4.3^a^	16.0 ± 1.2^a^	23.7 ± 5.8^a^	24.3	0.09
4	Total thickness (mm)	H	25.4 ± 3.8^a^	26.1 ± 6.0^a^	28.4 ± 10.4^a^	27.4	0.85
		V	21.8 ± 5.2^a^	22.8 ± 1.1^a^	30.2 ± 5.7^a^	29.8	0.10
5	Muscle intensity (a.u.)	H	0.6 ± 0.1^a^	0.7 ± 0.2^a^	0.7 ± 0.1^a^	0.3	0.42
		V	0.6 ± 0.1^a^	0.7 ± 0.1^b^	0.6 ± 0.0^a^	0.4	0.04

Description: The data were presented in the form of means with standard deviation (mean ± SD), different letters of superscript (^a,b^) on the same line show significant differences (*P* < 0.05), H = horizontal, V = vertical, TB = non-pregnant, B < 1 = Pregnant less than 1 month, B2–3 = 2–3 months pregnancy, PP = 2 weeks postpartum, *) not included in statistical calculation

**Fig. 2:**
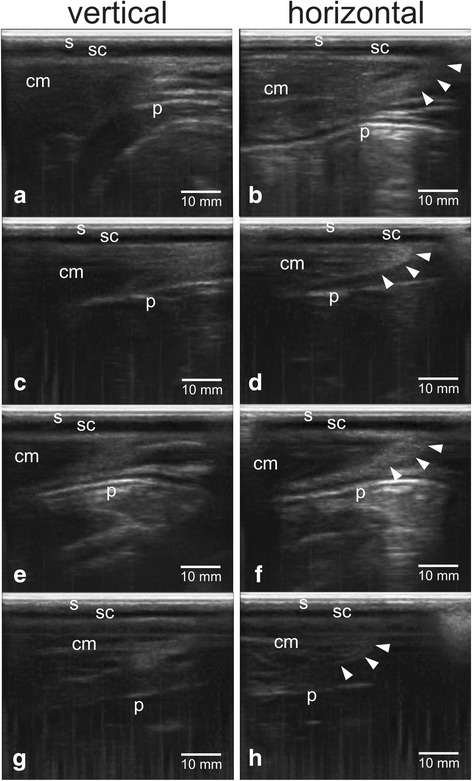
Sonogram of perineal tissue between sacrum and ischium bones of dairy cows with different gestational ages using transcutaneous B-mode ultrasonography: **a**, **b** non-pregnant, **c**, **d** <1 month pregnant, **e**, **f** 2–3 months pregnant, **g**, **h** postpartum, vertical scanning angles **a**, **c**, **e**, **g**, horizontal scanning angles **b**, **d**, **f**, **h**. Note: s = skin, sc = subcutaneous, cm = coccygeus muscle, p = peritoneum, arrow head = side part of coccygeus muscle

Figure 2 showed the confirmation results of perineal tissue between the sacrum and ischium bones with different gestational status of dairy cows using transrectal B-mode ultrasonography. Skin tissue (s) appeared as hyperechoic (white), subcutaneous tissue (sc) appeared as anechoic (black), muscle tissue (cm) appeared as hypoechoic (grey), while peritoneal tissue (p) appeared as hyperechoic.

**Fig. 3:**
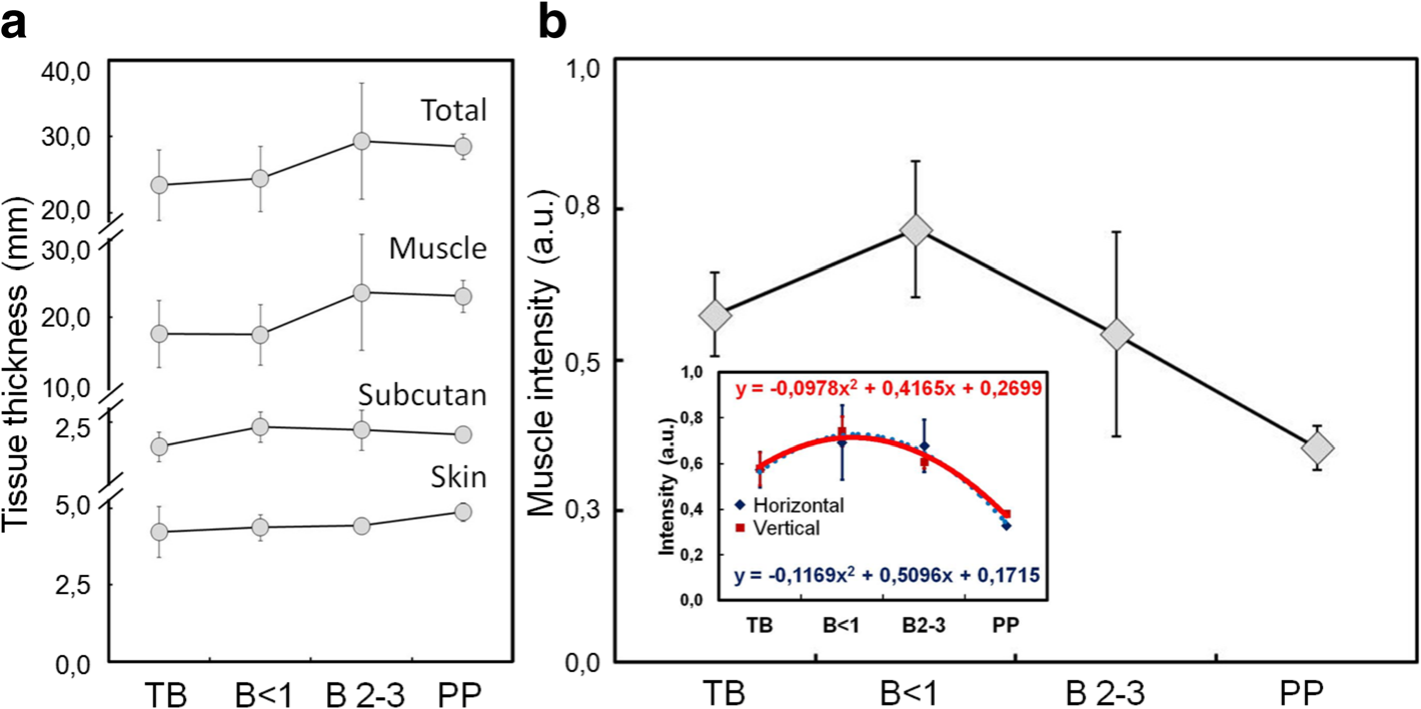
The average thickness of the perineal tissue between the sacrum and ischium bones (**a**) and the average intensity of coccygeus muscle (**b**) in dairy cows with different gestational ages and postpartum, (inset b) the normalized intensity of coccygeus muscle, horizontal scanning (*blue dashed line*, *R* ^*2*^ = 0.976) and vertical scanning (*red solid line*, *R* ^*2*^ = 0.969). TB = non-pregnant, B < 1 = pregnant less than 1 month, B2–3 = 2–3 months pregnancy, PP = 2 weeks postpartum

Figure 3 showed the average size of perineal tissues and intensity of the coccygeus muscle from horizontal and vertical viewpoints of sonogram images in dairy cows with different gestational status and postpartum. The tissue thickness tend to increase with gestational age and postpartum (Fig. 3a). However, the intensity of coccygeus muscle increased at young gestational age but subsequently decreased with the increase of gestational age and postpartum (Fig. 3b).

## Discussion

### Enclosure record of dairy cows

Enclosure record is one of medical records that can be used as a reference in the reproduction management of dairy cows. Dairy cows that have been inseminated and did not show symptoms of estrous after 21 days are considered pregnant. Gestational status examinations of AI results are performed generally at the pregnancy age of 2 months by perrectal palpation. The difference in the percentage of gestation from the enclosure record and the results of transrectal ultrasonography examination is reasonable because gestational failure might occur in livestock (Table 1 and Fig. 1). Gestational failure may occur due to improper detection of estrous [2], too early or late inseminations [11], reproductive diseases [12] or disorders [13], semen quality [14], metabolism [15] and also pregnancy loss [16]. Gestational failure as of pregnancy loss generally occurs in dairy cows at young gestation [17]. Pregnancy loss of 7% in dairy cows can be divided into 1.3% in the first trimester, 3.4% in the second trimester, and 2.3% in the third trimester [18]. Embryonic loss generally occur in the first 16 days of pregnancy, with the greatest incidence before 8 days post-conception in dairy cows with high milk production [16]. As a result, the gestation percentage of ultrasound confirmation is seen to be lower than that of the enclosure record.

### Changes in the anatomy of reproductive organs

Female reproductive organs during the reproductive cycle always undergo anatomical changes. The size and weight of the female reproductive organs are different in non-pregnant cows, estrous cows, gestation cows, postpartum cows or cows with disorder's reproductive conditions. These conditions can be identified by various obstetrics and gynecologic diagnostic techniques. Rectal palpation and ultrasonography are some of the techniques that can readily determine the changes that occur in the female reproductive organs. Rectal palpation can recognize a change in uterine size and the presence of fetal membrane slip in dairy cows at gestational age of >35 days [5]. Transrectal examination by ultrasonography can identify gestational sacs in the lumen of the uterus at gestational age of <2 weeks [19]. This research succeeded in another approach of imaging the status of reproductive organs indirectly through the measurement of perineal tissue size and intensity of coccygeus muscle using transcutaneous ultrasonography (Figs. 1, 2, and 3). Analysis of the size of the perineal area constituent tissue showed differences between different gestational ages and several days of postpartum (puerperium period) (Table 2). The uterus at young gestational age continued to grow larger and heavier in accordance with the increase of gestational age. Change in weight and size also occur during postpartum where the fetus has been removed from the uterus, causing a drastic weight loss of organs [20]. The puerperium period involves the process of uterine involution, where the change in uterus size and weight decreases from approximately 10 kg (± 100 cm length) right after partus back to the normal size of approximately 0.8 kg (± 25 cm length) [21].

### Constituent tissue of perineal area

The skin is a soft tissue that wraps the body and its underside as a border with surrounding muscles or tissues called subcutaneous [22]. The image size and intensity of skin in sonogram as well as subcutaneous tissue do not change in size and intensity with the gestational status, gestational age and postpartum (Table 2, Figs. 2 and 3). Anatomically, skeletal and muscular system in the perineal area are very complex [23]. Perineum is a fibromuscular tissue that acts as a border or pelvic diaphragm [24]. The perineal supporting bone comprises of the sacrum bone, coccygeus bone, and pelvic bone: consisting of coccy, ischium, ilium, and pubis bones [25]. Muscular system in the perineal area between the sacrum and ischium bones comprise of piriformis, coccygeus, iliococcygeus, and pubococcygeus muscles [9]. In ruminants, the apparent muscular tissue is the coccygeus muscle that forms the border between the sacrum and ilium bones. Coccygeus muscle is a muscle that originates from the spinal bone of ischium and the surface of the sacroisiatic ligament. This muscle extends and connects to part of the first 3 coccygeal bones and serves to move the tail with the nervous system from the pudendal nerve and the caudal rectus from the lower branches of the sacral nerve [26].

### **Ligaments of f**e**male reproductive organs**

The ligaments that support the female reproductive organs in the abdominal cavity consist of [27]: a) round ligament, b) broad ligament, c) suspensory ligament, and d) major ligaments: consisting of 1) major ligament, 2) uterosacral ligament, and 3) pubocervical ligament. The round ligament supports the uterus from the edge (parametric) of the uterine horn to the inguinal canal and links to the labia (vaginal opening) [28]. This ligament serves to maintain the uterus from torsion. Broad ligament is divided into mesovarium, mesosalpinx, and mesometrium where each of which serves to hang the ovaries, fallopian tubes, and uterus in place [29]. The broad ligament is formed from two layers of peritoneum that hangs up the uterus and attaches at the dorsolateral pelvic wall of the ilium region. Ligament of suspensory ovary (an infundibulopelvic ligament or abbreviated with IP) is a ligament that supports the ovaries. The IP ligament is formed from peritoneal folds extending from the ovaries to the pelvic wall [30]. The cardinal or Mackenrodt’s ligament [31] also known as lateral cervical ligament or transversal cervical ligament. This main ligament lies on broad ligament attached to the cervical wall and linked in the lateral portion of the pelvic wall (spina ischium) in obturatorius muscle fascia [32]. The uterosacral ligaments or recto-uterine ligaments are fibrous tissues linked between the uterus and the front of the sacrum bone [31]. The pubocervical ligament is a ligament linked between the edges of the cervical serous wall and the symphysis pubis [33].

The reproductive organs in dairy cows are also supported by the pelvic ligaments as of in humans [34]. The weakness of the pelvic ligaments may have an effect on the prevalence and the risk of labor complications [35]. Pathological changes in the reproductive organs in the abdominal cavity of dairy cows are generally diagnosed through the anatomical appearance of vulva. The non-symmetric and twisted vulva due to torsion [36], emerged out due to prolapsus and hernia [37] indicate the abnormalities in the reproductive organs. Changes in the position of the reproductive organs causing the ligaments to become unbalanced between their pairs, both of right and left. This can result in a difference in tension (contraction and relaxation) in the muscular tissues (muscles and ligaments). The occurrence of the reproductive disorders might also indirectly affect the size and the intensity of the perineum tissue structure, particularly the coccygeus muscle.

The female reproductive organs attach to the muscular system at the perineal membrane of the pelvic bone. The intensity of coccygeus muscle showed differences among dairy cows with different gestational status and postpartum (Fig. 3). As gestational age increases, the fetal size continues to increase appraching the end of gestation period, ready to be born (Fig. 1). Uterus size increases with the increase of fetal size. The large fetal size and weight affect the tissues that are attached to the ligaments of the reproductive organs. A long gestation period causes prolonged contraction of the coccygeus muscle, affecting its strength and thickness (Table 2). The ultrasound image of muscles in contracting human arm and abdomen showed a change in thickness of approximately 1–9% [38]. The measurement of pelvic muscle in gestation of 20 weeks in nullipara women showed a change in the strength of contraction and the size of the perineal muscle [39]. The muscle adjusts to changes that occur in the reproductive organs, causing the size (Table 2) and the intensity of sonogram image to alter as well (Fig. 3). A series of coccygeus, iliococcygeus, and pubococcygeus muscles form the diaphragm in the pelvis [24]. The pelvic diaphragm regulates the further stage of birth process. The muscles of the pelvic diaphragm will relax optimally to provide a fetal outlet during vaginal delivery. Fetus, placentas, and allantoic fluids are removed at birth so that the size and weight of the reproductive organs decrease rapidly. The residual of placenta and fluid in the uterus appear as a multi-echogenic image using B-mode ultrasonography [40]. Changes in the size of the hiatal levator muscle at gestational age of 12, 36 weeks and 6 months after normal and caesarean delivery in women showed a change in size at rest and contraction [41]. The ultrasound image during postpartum of coccygeus muscle in this study showed an increase of perineal muscle size and a decrease the intensity with the increase of gestational age and 2 weeks postpartum (Fig. 3).

### Estrous, intramuscular fat, reproductive disorders, and the influence of anesthesia

Estrous is a symptom of sexual arousal characterized by an enlargement of the size of the external reproductive organs, production of large amounts of mucus, and erection of internal organs [2]. Although there is no supporting data, changes in the size and weight of organs during estrous may affect the size and intensity of coccygeus muscle on ultrasound images. Furthermore, prolonged estrous disturbance (nymphomania) due to follicular cysts results in sterility hump [42]. Sterility hump is formed by prolonged contraction of the perineal muscle causing the muscles to become tense and larger, thus it is possible to affect the sonogram image of the perineal tissue. Furthermore, intramuscular fat [43] that appeared as hyperechoic in sonogram may also influence the intensity of tissue and the thickness of muscle [44]. In addition, reproductive disorders associated with the position, size, and weight of reproductive organs such as 1) position change due to the torsion [36, 45], 2) death of fetus in the uterus such as mummification and maceration, 3) the growth of tissue in the uterus such as neoplasia [46], and 4) accumulation of fluids in organs such as mucometra, haemometra, chronic hydrometra endometritis, and pyometra [46] may also affect perineal tissue size and intensity, especially the coccygeus muscle. The change in reproductive organs due to reproductive disorders also cause changes in position, size, and weight, possibly difficult to distinguish the sonogram image and perineal constituents tissue size among dairy cows during the gestation period. In addition, the anesthesia which affects muscle relaxation [47] and reproductive hormones [2] should be taken for consideration, since it may also affect the size of the coccygeal muscle, which in turn will affect imaging process. Gestational failure as of pregnancy loss at young gestational age [17] should also be noted during image processing.

## Conclusion

Imaging of ultrasonography on the perineal area between sacrum and the ischium bones of dairy cows can be performed transcutaneously with ease. Changes in the tissue thickness and intensity of the coccygeus muscle are affected by gestational age. Even though there were still a number of limitations, the technique and parameters in this study may be used as a new method of gestational detection in dairy cows.

## Abbreviations

*: Not included in statistical calculation
AI: Artificial Insemination
B < 1: Pregnant less than 1 month
B2–3: 2–3 months pregnancy
B-mode: Brightness mode
Cm: Coccygeus muscle
CT: Computed Tomography
Fm: Fetal membrane
Gs: Gestational sac
H: Horizontal
KUNAK: Kawasan Usaha Peternakan
Lc: Lochia
Lm: Lumen
P: Peritoneum
Pl: Placenta
PP: 2 weeks postpartum
S: Skin
Sc: Subcutaneous
SPR: Sentra Peternakan Rakyat
TB: Non pregnant
Ute: Uterus
V: Vertical
Vu: Bladder
